# Paleomagnetic recording fidelity of nonideal magnetic systems

**DOI:** 10.1002/2014GC005249

**Published:** 2014-06-05

**Authors:** Adrian R Muxworthy, David Krása, Wyn Williams, Trevor P Almeida

**Affiliations:** 1 Department of Earth Science and Engineering, Imperial College LondonLondon, UK; 2 European Research Council Executive AgencyBrussels, Belgium; 3 School of GeoSciences, University of EdinburghEdinburgh, UK

**Keywords:** thermoremanence, paleointensity, magnetite, thin films, pseudo-single domain

## Abstract

A suite of near-identical magnetite nanodot samples produced by electron-beam lithography have been used to test the thermomagnetic recording fidelity of particles in the 74–333 nm size range; the grain size range most commonly found in rocks. In addition to controlled grain size, the samples had identical particle spacings, meaning that intergrain magnetostatic interactions could be controlled. Their magnetic hysteresis parameters were indicative of particles thought not to be ideal magnetic recorders; however, the samples were found to be excellent thermomagnetic recorders of the magnetic field direction. They were also found to be relatively good recorders of the field intensity in a standard paleointensity experiment. The samples' intensities were all within ∼15% of the expected answer and the mean of the samples within 3% of the actual field. These nonideal magnetic systems have been shown to be reliable records of the geomagnetic field in terms of both direction and intensity even though their magnetic hysteresis characteristics indicate less than ideal magnetic grains.

**Key Points:**

- Nonideal magnetic systems accurately record field direction
- Weak-field remanences more stable than strong-field remanences

## 1. Introduction

Extracting the directional information recorded by natural remanent magnetizations (NRM) of thermal origin has long been shown to be relatively reliable regardless of the magnetic domain state of the particles within a rock [*Ozima and Ozima*, 1965]; however, recovering the intensity of the field imparting this magnetization is less straightforward. Most methods of recovering the absolute ancient field intensity (paleointensity) rely on stepwise replacing the NRM acquired by an igneous rock on cooling with a laboratory analogue, i.e., a thermoremanence (TRM) [*Coe*, 1967; *Thellier and Thellier*, 1959]. These time-consuming methods have met with mixed success: there are many reported cases with very high failure rates, as high as 100% in some cases [*Paterson et al*., 2010]. There have been many attempts to modify the original methods or develop new ones [*Dekkers and Böhnel*, 2006; *Muxworthy and Heslop*, 2011], but in nearly all cases the underlying theory upon which these methods are based, i.e., for particles with uniform magnetizations termed single domain (SD), is not strictly applicable to the magnetic domain states commonly found in natural samples, i.e., grains with nonuniform magnetic structures that are termed pseudo-SD (PSD) or multidomain (MD). Current theoretical understanding of the recording fidelity of PSD TRM is poor due to the highly nonlinear nature of the problem: There are currently no analytical or numerical models which accurately explain the thermomagnetic behavior of such grains.

**Figure 1:**
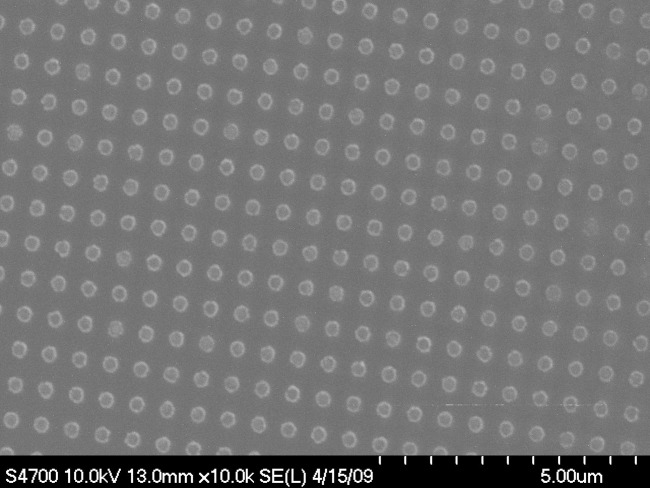
Scanning electron microscope image of sample DK0131.

One practical way to resolve this problem is to experimentally quantify the behavior of PSD TRM, however, the experimental investigation of PSD behavior has its own set of problems: The geometry and size of a magnetic crystal strongly controls its magnetic properties, as does its spatial relationship with respect to other magnetic particles [*Evans et al*., 2006; *Muxworthy et al*., 2003]. In order to systematically examine the influence of these parameters on the magnetic properties in general and the stability and fidelity of the magnetic recording in particular, we need to study samples with well-defined and controlled physical characteristics. Natural systems of magnetic minerals in rocks will almost always contain a broad range of particle size, shape, and spacings, and so we need to use synthetic systems. Most methods of synthesis produce samples with wide grain size distributions and variable spatial distributions, e.g., hydrothermal methods [*Heider and Bryndzia*, 1987]. However, electron-beam lithography (EBL) produces thin films of two-dimensional arrays of magnetic mineral particle assemblages with very well-defined composition and particle geometry [*King et al*., 1996]. In a previous study [*Krása et al*., 2009], we described the nanofabrication of arrays of magnetite crystals using EBL (Figure 1), and in *Krása et al*. [2011] reported the room-temperature and low-temperature properties of 10 EBL samples. The samples contain equidimensional grains with a range of sizes from 74 nm up to 333 nm [*Krása et al*., 2011], and a range of controlled interparticle spacings, which are thought to be both interacting and noninteracting [*Muxworthy et al*., 2003]. In this paper, we report high-temperature measurements, including a synthetic paleointensity investigation, to help us understand the thermomagnetic recording fidelity of PSD particles.

## 2. Samples and Methods

The nanofabrication technique used in this study to make the new samples has been described extensively by *Krása et al*. [2009]. The samples' physical, room-temperature hysteresis properties, and Verwey temperatures were reported previously in *Krása et al*. [2011] and are summarized in Table1. For consistency, we use the same sample names as used in previous papers, and due to the two-dimensional nature of the samples, both the in-plane and out-of-plane measurements are referred to.

**Table 1:** Physical and Bulk Magnetic Parameters for the Samples Considered in This Study^a^

Sample	Dot Diameter (nm)	Separation (nm)^b^	Dot Height (nm)	Orientation	*M*_RS_*/M*_S_	*H*_C_ (mT)	*H*_CR_ (mT)	*H*_CR_/*H*_C_	*T_v_* (K)	*TRM/SIRM* (%)
DK0011^c^	265	310	192	In-plane	0.35	23	38	1.66	110	2.8
				Out-plane	0.16	14	46	3.25	113	…
DK0023^c^	100	310	102	In-plane	0.29	17	33	1.98	114	1.3
				Out-plane	0.17	13	35	2.80	…	…
DK0024-2^c^	120	180	102	In-plane	0.55	31	42	1.34	100	2.0
				Out-plane	0.17	27	136	5.11	…	…
DK0034	281	310	102	In-plane	0.38	30	50	1.67	114	2.0
				Out-plane	0.17	18	86	4.90	110	…
DK0121	243	600	39	In-plane	0.21	14	40	2.83	119	1.2
				Out-plane	0.14	11	29	2.74	…	…
DK0124right	74	300	39	In-plane	0.11	5.2	17	3.33	…^d^	1.3
				Out-plane	0.09	5.1	17	3.25	…	…
DK0127	“Wide”	200	65	In-plane	0.14	6.5	22	3.40	…^3^	1.0
				Out-plane	0.10	5.5	19	3.37	…	…
DK0131	333	600	65	In-plane	0.28	15	48	3.23	110	3.5
				Out-plane	0.17	13	46	3.50	…	…
DK133-1	229	600	65	In-plane	0.16	7.5	29	3.93	…	2.0
				Out-plane	0.08	5.5	26	4.70	…	…
DK133-8	178	300	65	In-plane	0.12	5.1	18	3.61	…	1.1
				Out-plane	0.10	5.8	19	3.19	…	…

a Some of the magnetic parameters were reported previously in *Krása et al*. [2009,2011], and given here for completeness. The TRM in the final column was induced in a field of 60 µT.

b This is the grain center-to-center separation, e.g., sample DK0024-2 has a particle edge separation of 60 nm.

c Samples previously reported by *Krása et al*. [2009].

d Measured but Verwey transition temperature not clearly identified.

Most of the samples are in the middle of the PSD range [*Muxworthy and Williams*, 2006], with a range of intergrain spacings, ranging from what are thought from numerical models [*Muxworthy et al*., 2003] to be noninteracting, e.g., DK0121, to arrays of magnetite that are likely interacting, e.g., DK0011 (Table1). Given its dot size and interdot spacing, sample DK0124right is likely noninteracting and may possibly be SD as its size resides on the SD/PSD boundary [*Muxworthy and Williams*, 2006]; however, its hysteresis parameters (Table1), suggest that its magnetization is nonuniform, i.e., in PSD state. For samples with clearly identifiable Verwey transitions, the temperatures of this transition were close to that of stoichiometric magnetite [*Muxworthy and McClelland*, 2000]. Sample DK0124right did display anomalous behavior in the range 100–130 K, but no clear Verwey temperature was identified [*Krása et al*., 2011]. In very small grains, i.e., like DK0124right, the Verwey transition is often suppressed. Sample DK0127 did not display any transition behavior at low temperatures [*Krása et al*., 2011], suggesting that it may not have been stoichiometric magnetite.

The thermoremanence measurements reported in this paper were all conducted at the Kochi Core Center, Kochi University, Japan, using a combination of a Natsurhara-Giken TDS-1 paleomagnetic oven and a single-sample 2G magnetometer. For normalization purposes, a saturation isothermal magnetization (SIRM) was induced in a field of 1 T using a Magnetic Measurements MMPM10 pulse magnetizer.

Before making the measurements the samples were vacuum-sealed in quartz-glass capsules. The samples were fixed to the inside of the capsules using Omega CC high-temperature cement. Before vacuum sealing, the samples had been stored in alcohol since last reduced.

## 3. Results

### 3.1. SIRM and Thermoremanence Measurements

The samples were induced with a SIRM using a field of 1 T, followed by a TRM in a field of 60 µT on cooling from 650°C. Due to the shape of the quartz capsules both the SIRM and TRM could only be induced in the plane of the nanodots unlike the experiments reported by *Krása et al*. [2011] who measured both in-plane and out-of-plane remanences. The ratio of TRM/SIRM is shown in Table1. It is seen that as the dot size increases, the TRM/SIRM ratio increases.

### 3.2. TRM Thermal Demagnetization

**Figure 2:**
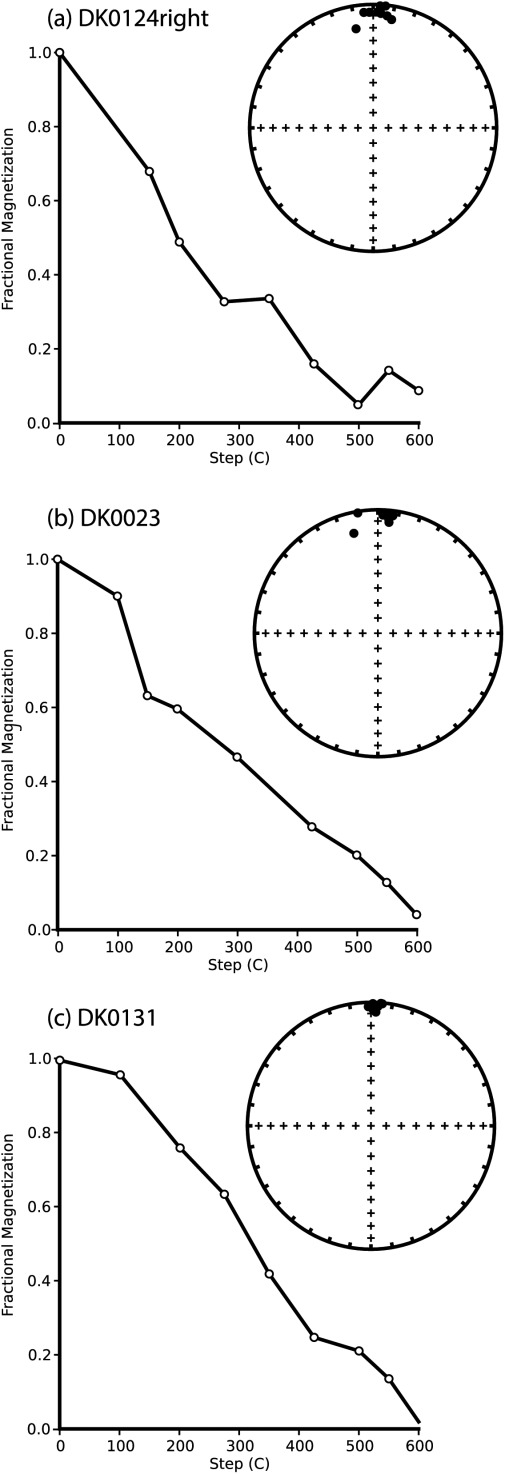
Stepwise thermal demagnetization data for samples: (a) DK0124right, (b) DK0023, and (c) DK0131. On the left-hand side, the TRM intensity is plotted as a function of temperature. On the right-hand side, the direction of the TRM during thermal demagnetization is plotted on an equal-area projection plots. The TRM was induced in a field of 60 µT.

The samples' in-plane TRMs were then stepwise thermally demagnetized (Figure 2). The measured TRM unblocking spectra are relatively coarse; however generally, the samples demagnetize gradually, displaying wide unblocking spectra. The TRM direction displayed in the equal-area projection plots was very stable during thermal demagnetization, and aligned with the inducing field.

### 3.3. Paleointensity Results

After the initial TRM experiments, six of the magnetically stronger samples were selected for a synthetic paleointensity study: DK0011, DK0023, DK0024-2, DK0124right, DK0127, and DK0131. The samples were first induced with a TRM in a field of 100 µT (the “NRM”), and a paleointensity study conducted following the standard double-heating protocol of *Coe* [1967], with pTRM checks, pTRM-tail checks [*Riisager and Riisager*, 2001; *Walton*, 1984], and pTRM additivity checks [*Krása et al*., 2003]. For the paleointensity determination, a laboratory field of 100 μT was applied parallel to the NRM during both heating and cooling cycles for each in-field treatment. Sixteen heating steps were made between 75 and 600°C combined with seven pTRM checks, seven pTRM-tail checks, and two pTRM additivity checks.

**Figure 3:**
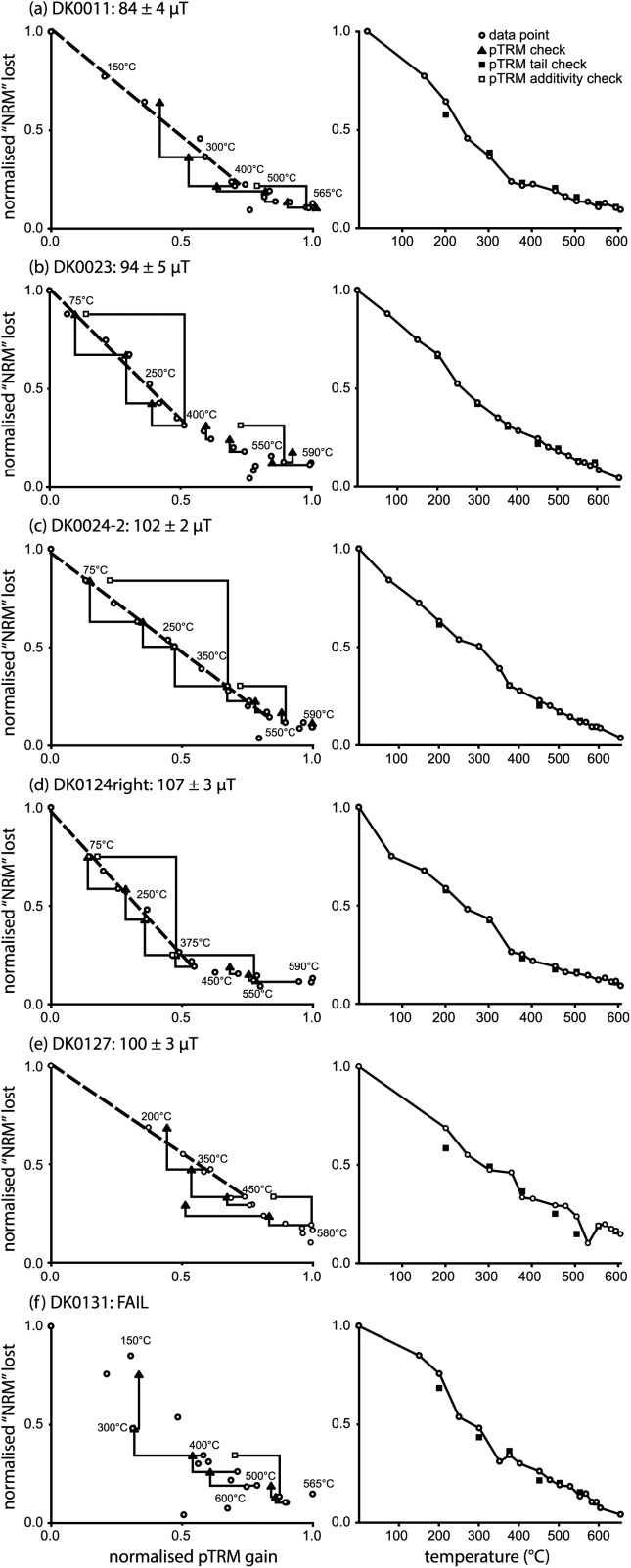
Paleointensity data for the six samples examined in the Thellier experiment: (a) DK0011 (NRM = 2.4 × 10^−9^ A m^2^), (b) DK0023 (NRM = 2.9 × 10^−9^ A m^2^), (c) DK0024-2 (NRM = 2.4 × 10^−9^ A m^2^), (d) DK0124right (NRM = 5.2 × 10^−9^ A m^2^), (e) DK0127 (NRM = 1.4 × 10^−9^ A m^2^), and (f) DK0131 (NRM = 1.8 × 10^−9^ A m^2^). The NRM for the experiment was a TRM induced in a field of 100 µT. On the left-hand side Arai plots with pTRM checks and pTRM-additivity checks are shown, and on the right-hand side the NRM demagnetization curves with corresponding pTRM-tail checks. On the Arai plots, paleointensity estimates were made using the selection criteria detailed in Appendix (Table A1). Sample DK0131 failed to pass these criteria.

Arai plots with corresponding NRM demagnetization plots are shown for all the samples in Figure 3. The Arai plots display relatively linear behavior up until ∼400°C. Afterward there is some scatter in the Arai plots, though the thermal demagnetization plots derived from the initial TRM component displays consistent demagnetization trends (Figure 3, right-hand side). There are two likely reasons for this scatter or “noise” at high temperatures: (1) the low magnetic strength of the samples, meant that at high temperatures the signal-to-noise was low for the NRM demagnetization steps as the sensitivity limits of the instrument were approached, and (2) chemical alteration for which there was some evidence even though samples were vacuum-sealed in quartz-glass capsules. This chemical alteration was highlighted by the lack of pTRM check repeatability (Figure 3), and physically in a slight discoloration of the inside of the quartz-glass capsules. The degree of discoloration increased as the paleointensity experiment progressed to higher temperatures. It is suggested that this visible alteration may have been due to the silicate substrate altering; the samples had been annealed on several occasions, which involves heating to 600°C [*Krása et al*., 2001,2009], so it was expected before the experiment that the samples would be thermally stable in a vacuum at these temperatures.

The results were analyzed with the ThellierTool (v. 4.22) software of *Leonhardt et al*. [2004] (Table2). ThellierTool's default selection criteria (see Appendix Table A1) were used to classify the results, which were determined by maximizing the quality factor (*q*). Five out of the six samples passed the selection criteria; sample DK0131 (Figure 3f) failed to yield a reliable intensity estimate. The five successful samples provided paleointensity estimates that were within 16% of the expected value of 100 µT (Table2). Most of the estimates were from relatively low temperatures, though this reflects the poor quality of the Arai plots at high temperatures. There is one possible exception to this, sample DK0124right. It displayed curvature in its Arai plot (Figure 3d), and its curvature parameter value (*k*) [*Paterson*, 2011] was relatively high (Table2). The paleointensity selected for this sample was also the highest, which could be the result of picking from the steeper side of a curved Arai plot.

**Table 2:** The Thellier Results Measured for This Study^a^

Sample	Intensity (µT)	±σ (µT)	ΔT (°C)^b^	*N*	*f*	*FRAC*	*g*	*q*	*w*	*k*	*δ (CK)*	*δ (TR)*	*δ (AC)*	Class
DK0011	84	4	20–375	7	0.77	0.70	0.78	12	5.2	0.36	7	6.7	…^c^	B
DK0023	94	5	20–375	8	0.68	0.65	0.84	10	4.2	0.60	4.1	1	9.8	B
DK0024-2	102	2	20–525	13	0.86	0.88	0.89	47	14	0.24	5.5	2.7	9.5	B
DK0124right	107	3	20–450	10	0.81	0.81	0.82	22	7.8	0.95	4.3	1.8	4.5	A
DK0127	100	3	20–375	6	0.66	0.52	0.67	15	7.4	0.11	6.8	10.3	…^c^	B

a Six samples were studied; sample DK0131 failed to yield a recoverable intensity value. Definitions of the various parameters are provided in Table 3, including the class.

b Δ*T* is the temperature range used to make the paleointensity estimate.

c Measured, but not used in the final intensity calculation as outwith Δ*T*.

## 4. Discussion

During thermal demagnetization of the samples' thermoremanences, all the samples were found to be reliable recorders of the inducing-field direction (Figure 2), i.e., samples which are “nonideal” recorders appear to reliably retain the magnetizing field direction. This finding supports the conclusions of *Krása et al*. [2011] who found for the same samples that anhysteretic remanent magnetizations (ARMs)—low-field remanences—were directionally more stable during demagnetization than SIRMs—high-field remanences. *Krása et al*. [2011] found the contrast in stabilities was greater for the out-of-plane measurements; only in-plane measurements were made in this study due to physical constraints, however, the new data still supports previous findings.

**Figure 4:**
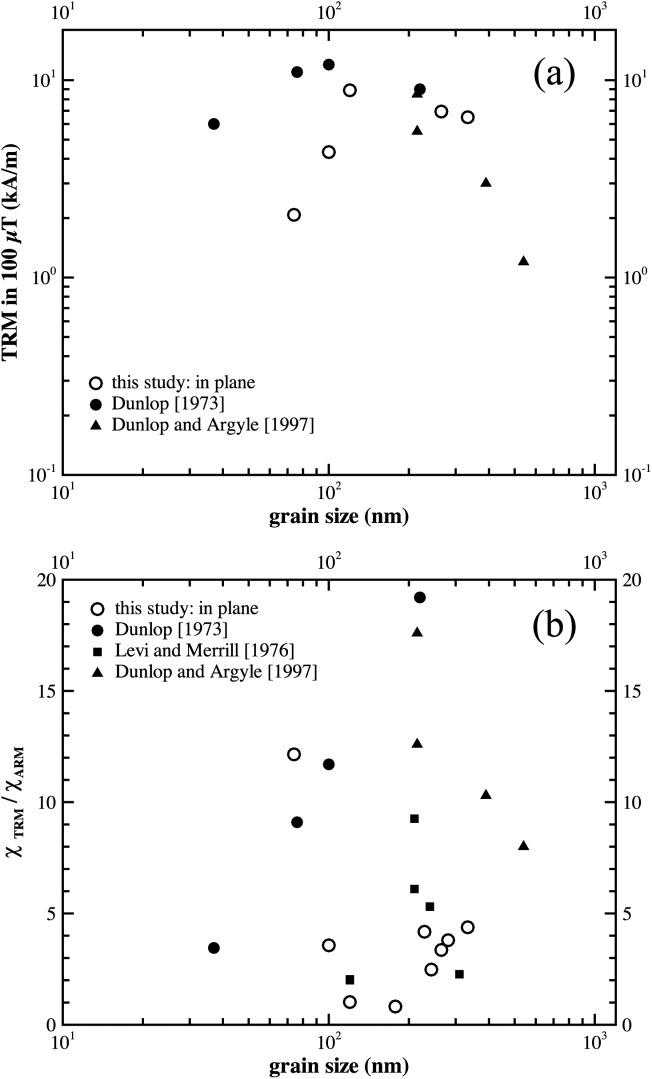
(a) Plot of TRM intensity induced in a field of 100 µT versus dot size. The data of *Dunlop* [1973] and *Dunlop and Argyle* [1997] are for synthetic hydrothermally grown, magnetic powders. The TRM intensities are estimated for the EBL samples from the SIRM values and the hysteresis ratios (Table1), and the assumption that the samples are pure magnetite. (b) Ratio of the susceptibility of TRM (χ_TRM_) over ARM (χ_ARM_) for EBL samples in this study, plus the hydrothermally grown, synthetic powders of *Dunlop* [1973] and *Dunlop and Argyle* [1997], plus crushed, sized, magnetite powders, or *Levi and Merrill* [1976].

The samples displayed thermoremanence intensities that were between 1.0 and 3.5% of their SIRM values (Table1). To compare the data with previously published data for thermoremanence induced in sized, powdered magnetite samples in a field of 100 µT, we normalize the TRM intensity by the mass of the samples. As the exact number of dots is not known we use the TRM/SIRM and *M_RS_/M_S_* ratios (Table1) with the assumption that the crystals are stoichiometric magnetite and thus have a spontaneous magnetization of 480 kA/m [*Pauthenet and Bochirol*, 1951], to make an estimate for TRM in kA/m (Figure 4a). It is seen that the trend of the EBL data is significantly different to the published data for hydrothermally produced samples (Figure 4a): As the grain size decreases below 100 nm the TRM intensity drops more sharply for the EBL samples. From their hysteresis parameters (Table1) none of the samples display ideal SD behavior, i.e., they appear to contain PSD states that are likely to be vortex domain states. As the grain size decreases below 100 nm, the reduction in TRM probably reflects the reduction in the absolute size of the vortex-core size and the associated core moment, that is, the decrease in TRM is due to the nanodots containing smaller vortex-core moments.

As the calculation of TRM per unit mass relies on a number of assumptions, we also plot the susceptibility of TRM (χ_TRM_) over the susceptibility of ARM (χ_ARM_) (Figure 4b). The ARM data were reported in *Krása et al*. [2011]. For the EBL samples, the ratios are quite low compared to the published data, plotting closer to the crushed magnetite than that of the hydrothermally grown samples, with the exception of DK0124right (Figure 4). That χ_TRM_/χ_ARM_ is mostly >1, reflects the ability for some particles to become blocked in higher remanence states during TRM acquisition compared to the ARM state. The relatively high ratio for DK0124right suggests that this effect is most pronounced in smaller particles. Such states are unattainable to the same particles during ARM induction (or is less likely), and they are again less likely for the larger particles during TRM acquisition.

These differences between the EBL, crushed and hydrothermally grown samples shown in Figure 4, probably reflects the very narrow grain size range of the EBL samples, i.e., the TRM signal of powdered samples with similar nominal grain sizes (Figure 4a) is influenced by larger (or smaller) crystals. However, the differences between the sample origins may also reflect variable levels of internal stress; the stress in the EBL samples arising from crystal lattice mismatches at the substrate/crystal interface.

**Figure 5:**
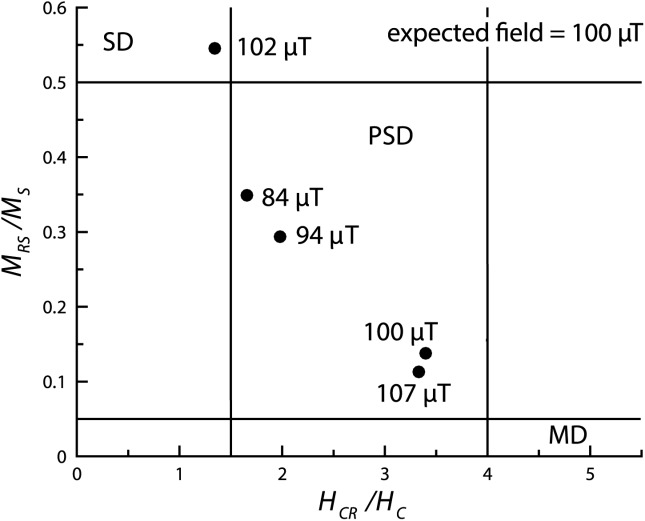
A “Day plot” [*Day et al*., 1977] of the ratios of the hysteresis parameters *M_RS_/M_S_* versus *H_CR_/H_C_* for the five EBL samples listed in Table2 that yielded intensity estimates. The intensity estimates and the regions commonly associated with SD, PSD, and MD behavior are labeled.

The samples displayed wide TRM unblocking spectra and hysteresis behavior that are more akin to MD behavior (Figure 2 and Table1), however, they returned paleointensity Arai plots that were relatively linear, albeit over the lower temperature range. At higher temperatures, the Arai plots became increasingly noisy. Five out of six paleointensity determinations passed the selection criteria; of this five, four yielded estimates with 7% of the applied field with the other sample provided an estimate that was 16% too low. The paleointensity estimates were also relatively independent of grain size and domain state as indicated in Figure 5 on a “Day” plot [*Day et al*., 1977], and were all close (∼15%) to the expected value of 100 µT. The mean of the five samples was 97 ± 8 µT, which corresponds to a 95% confidence interval of 90–105 µT. This suggests that the grains with magnetic domain states in the PSD range, i.e., particles that carry relatively simple vortex structures, can yield successful paleointensity estimates. It also supports previous reports [*Carvallo et al*., 2006; *Krása et al*., 2011] that suggest preselection for paleointensity determination via high-field measurements may not be indicative of low-field thermoremanence behavior.

There are a number of factors that question the universality of the laboratory paleointensity study in this paper. First, for example, there is uncertainty in the magnitude of the effect of aligning the TRM field direction with the initial NRM direction in the paleointensity experiment on the final intensity estimate, though it is likely it would improve the accuracy. In future studies, it would be worth repeating these experiments for a range of different angles, unfortunately, that was outside the scope of this study as there was evidence that the samples may have chemically altered during the first paleointensity experiment. Second, the cooling rate for these samples was the same in both the NRM acquisition and TRM acquisition. Future laboratory experiments could investigate this, but it is difficult to generate geologically comparable long cooling times in the laboratory. Certainly, the viscous decay of TRM in such samples should be investigated in the future.

## 5. Conclusions

Ten samples produced by electron-beam lithography with near-identical grains in the pseudo-single domain size range have been induced with thermoremanences, and their thermomagnetic properties examined including their ability to record reliable paleointensity information. They were found to be reliable recorders of both the intensity and direction of the geomagnetic field. On comparison with *Krása et al*. [2011] it is seen that high-field remanences, i.e., saturation isothermal remanences, can be unstable in such samples, but these domain states appear to be much better at recording low-field remanences like TRM and room-temperature anhysteretic remanent magnetizations (ARM). It is suggested that the use of high-field measurements to preselect samples for paleointensity determination may be flawed.

## Appendix A

In Table A1 the paleointensity parameters referred to in the manuscript are defined. In addition to the definitions, in Table A1 the default selection criteria for ThellierTool 4.22 [Leonhardt et al., 2004] are given.

**Table A1:** Summarizing the Paleointensity Parameters Used in Table1^a^

Criteria Description	Class A	Class B
*Linear Fit Criteria*		
Number of points (*N*) used to determine the paleointensity	≥5	≥5
Normalized standard deviation of slope (*β*)	≤0.1	≤0.15
Fraction of NRM (*f*)	≥0.3	≥0.3
Quality factor (*q*)	≥1	≥0
*Directional Criteria*		
Maximum angular deviation (MAD) of the anchored fit	≤6°	≤15°
Angular difference between the anchored and nonanchored solution (*α*)	≤15°	≤15°
*Alteration Criteria*		
Maximum difference produced by a pTRM check, normalized by the TRM (δ(CK))	≤5%	≤7%
Cumulative difference produced by pTRM checks (δ*_pal_*)	≤5%	≤10%
*Repeated Demagnetization Steps*		
Extent of pTRM tail after correction for angular dependence pTRM (δ(t*))	≤3%	≤5%
Maximum difference produced by a pTRM-tail check, normalized by the NRM (δ(TR))	≤10%	≤20%
*Additivity Checks*		
Cumulative difference produced by pTRM additivity checks (δ(AC))	≤3%	≤5%
*Additional Parameters*		
The curvature of the Arai plot as determined by the best fit circle to all of the data (*k*)	···^b^	···^b^
*FRAC*: the vector difference sum (VDS) of the selected component divided by the total VDS	···^b^	···^b^

a These parameters are described in more detail in *Leonhardt et al*. [2004], *Paterson* [2011], and *Shaar and Tauxe* [2013], and references therein. The ThellierTool 4.22 default criteria are defined for class A and class B; the limits are listed below. Common abbreviations for the symbols are bracketed.

b Parameters not included in ThellierTool criteria.
